# Ethics in the COVID-19 pandemic: myths, false dilemmas, and moral overload

**DOI:** 10.1007/s10676-020-09568-6

**Published:** 2021-03-12

**Authors:** Georgy Ishmaev, Matthew Dennis, M. Jeroen van den Hoven

**Affiliations:** TU Delft, Delft, The Netherlands

The global push to utilise mobile technologies in the fight against the COVID-19 pandemic has caused an unprecedented spurt of engineering. The accelerated development and deployment of these technologies has resulted in technical solutions that have completed a full innovation cycle—from speculative proposal to abandoned project—in a few frenetic months^1^. Such accelerated innovation is proving to be costly; it is also rife with ethical pitfalls. Both public and private actors find themselves confronted with a lack of accurate data, chronic uncertainty, or complete ignorance when trying to deal with the pandemic’s multifarious and shifting challenges. Simultaneously, there is an urgent need to deal with a veritable phantasmagoria of ethical dilemmas, value conflicts, and moral disagreements. In striving to accommodate multiple values, obligations, duties, and responsibilities, decision makers at all levels have reached what has been termed ‘moral overload’ (van den Hoven et al. 2012). This describes a situation in which a moral agent is unable to meet their obligations and ethical responsibilities. Privacy and confidentiality are important, but so are transparency and accountability. Health and public safety are vital, but so are social interaction, education, and jobs. How can we ensure that we have both? Is it naïve to hope that we still may be able to have all? These questions have confronted (and often confounded) both ethicists and laypersons.

Governments and authorities from around the world have been willing to bite the bullet when confronted by these situations and to take drastic measures: imposing restrictions on movement, prohibiting gatherings, derogating from fundamental human rights, and discarding privacy protections in order to serve public health or the economy.^2^ We believe that this is problematic—for the reasons we discuss below—and that it is alarming in the context of profound uncertainty regarding the efficiency of the imposed measures and employed technological tools.

It is right to worry that overconfidence in, and overreliance on, technological solutions could detract from the other critical tools of epidemiology, and provide a false sense of security. Speculative technological ‘silver bullets’ should not be seen as a replacement for proven tools in fighting a pandemic—such as manual contact tracing, personal protective equipment, widespread testing, and other preventive measures. This means that an ethics of information technologies in this context should not focus on the micro level and on digital innovations and components in isolation. Rather, we contend, its level of analysis should be technological ecosystems and socio-technical systems, viewing these in the context of the overarching ‘systems of systems’ that these systems comprise.

Such a perspective exposes major ethical challenges concerning the proportionality of the deployment of proposed technological tools. The design of these tools needs to be informed first and foremost by the aims of clinical medicine and public health. One of the key difficulties is that our capacity to meet these ethical requirements can be hampered by overly focusing on emergencies and anomalies that bear down on us today, at the expense of systematic and diachronic considerations.

The deployment and repurposing of surveillance technology is particularly worrying from this perspective, even in countries with a strong rule of law and institutional privacy protections. The systems perspective highlights that while the existing legal standards of data protection can provide some privacy assurances, they cannot address all ethical issues that are raised by the deployment of health surveillance technology.^3^ More specifically these issues require careful attention:

I. *Proportionality* Legal compliance of proposed digital tools with privacy regulations, does not in and by itself address the question of proportionality in the absence of evidence on the efficacy of those tools.
II. *Function creep* There is a strong potential for ‘function creep’, when collected private data is used for other purposes other those initially claimed.
III. *Sunset clauses* There is a risk that so-called ‘sunset clauses’ of emergency surveillance are ignored, and these capabilities stay in place after the crisis.
IV. *Non-voluntariness* If tools are used only on a strict voluntary basis, there is a risk that other emerging (economic and social) incentives can make them *de-facto* obligatory.

These issue are underscored by the rapid normalisation of numerous surveillance tools, a state of affairs that would have been considered unthinkable only a few months ago. Surveillance bracelets—previously used only in the criminal detention contexts—are now used to track quarantined individuals.^4^ Digital medical certificates and facial recognition technology are starting to be discussed enthusiastically as necessary preconditions to enjoy a future social life.^5^ Health smartphone apps—initially envisioned as informational tools for individuals—become repurposed into social control tools, used to segregate people into colour-coded categories, according to their ‘degree of uprightness and diligence in carrying out party work’.^6^

From the beginning of the crisis, smartphone apps have been at the centre of public discussion of COVID-19 technologies. This can be explained by the fact that smartphones are now ubiquitous across many populations, and have broad sensor and connectivity capabilities that enable a wide range of health surveillance functionality. The collection of private data in this context allegedly serves two main purposes: (1) slowing the spread of infection, informing the users on risks and nudging them into preventive measures (social distancing, quarantine, etc.), and (2) providing medical researchers and authorities with potentially vital epidemiological data. Furthermore, initially the ubiquity of smartphones was viewed as a way to accelerate adoption of app based tools on a mass scale. This assumption so far has turned out to be overly-optimistic. It ignores other critical success factors for the adoption of these tools: privacy, the trust and compliance of users, and integrating the app with traditional epidemiological tools (such as testing).^7^

Currently, various app-based solutions are at different stages in the innovation cycle—ranging from early implementation proposals to abandoned projects. Many of these discarded projects present a sobering illustration on the complexity of deploying app-based tools. In the UK, a nationwide attempt to combine multiple functionality of epidemic tracking, contact-tracing, and algorithmic symptom evaluation in one package quickly ran into trouble.^8^ Problems included data security, low efficacy, and the poorly defined functionality of app, especially when evaluated in relation to other pandemic containment tools and mechanisms. Quarantine app by Korean government was found to be implemented with major security flaws.^9^ Similarly, many apps built around centralised data collection were simply abandoned on the grounds of privacy, such as those developed by Norway, Lithuania, and Germany.^10^

In Europe, in terms of public uptake, narrowly purposed ‘exposure notification’ apps have been notably more successful. Facilitated by Apple’s and Google’s OS updates, these apps collect a minimal amount of Bluetooth sensor data for the purposes of notifying its users about whether they have been in close proximity to infected person. Notably, this approach eschews centralised collection of data. In Asia, despite its initial success using contact-tracing apps, Singapore has even seemingly shifted hopes away from apps altogether, and now favors contact-tracing wearables based on Bluetooth enabled ‘exposure notification’.^11^

Nevertheless, the idea of ‘super apps’ combining wide range of functionality ranging from symptom-checking to surveillance of infected patients to food delivery for quarantined patients has not been abandoned (Ferretti et al. 2020; Zastrow 2020). Pandemic-related ‘super apps’ have gained a strong foothold in China. From early in the pandemic, these apps have integrated data collection, quarantine enforcement, and police surveillance.^12^ In the US, the drive for ‘super-apps’ has shifted to the context of work place surveillance, where there are growing signs that it may become de-facto mandatory, as a condition of employment in some companies.^13^ Furthermore, some educational institutions in US seem to adopt Chinese style ‘super apps’ with mandatory location tracking and colour codes.^14^

Now that dealing with the pandemic has moved from initial shock crisis phase to various concerted attempts to lift quarantines and travel restrictions, various health surveillance tools risk becoming a permanent fixture.^15^ In fact many have explicitly been presented as a condition of 'returning to normal’.^16^ It is critical, therefore, to consider not only alternative technological solutions, but also path dependencies that will come to define these developments. Many health surveillance applications that have now been marketed as alternatives to lockdown measures, seem to embrace many of the false dilemmas we began this article discussing: “health vs privacy”, “health vs economy”, etc. For this reason, we believe that critical scrutiny is needed to avoid thinking of pandemic preventions technologies in these terms. This is supported by (the now numerous) examples of discarded contact-tracing apps, and other so-called ‘COVID-19 solutions’. These technologies need to be designed without false dilemma framing. So what are false dilemmas? And how can we avoid them?

## False dilemmas

Moral dilemmas cause moral overload in the agent who is confronted by a choice between incompatible values or obligations. Individuals and organizations can find it impossible to honour all their obligations (flatten the curve of COVID-19 infections *and* prevent unemployment rates to soar; trace all infected citizens while *also* respecting their privacy). Not all cases of moral overload present situations in which it is really impossible to escape the horns of the dilemma. There are sometimes creative or innovative solutions to what initially presents itself as a dilemma or is presented as a dilemma to us by others. In fact, often moral conflicts arrive because of bad decisions that have been made in the immediate past, decisions that could have prevented the dilemma from occurring if they had been made differently. One tragedy of the current pandemic is that dilemmatic situations can occur in many contexts simultaneously, forcing politicians and decision makers to take action, without them having an opportunity to prevent the dilemmatic situation from arising in the first place. Often governments have taken measures in the past that make it more likely that dilemmas would occur under pandemic conditions, e.g. by cutting the budgets in healthcare.^17^

Clear examples of such moral failure occur when institutionally embedded agents act for the sake of demonstrating readiness to act without scientific or other epistemic justification for this action. Sadly, the current crisis has already demonstrated numerous instances of such failure, ranging from flawed public-safety advice by politicians, to the deployment of technical solutions of questionable efficacy. And while the failure of a former type might be more flagrant, the development of some health surveillance systems has resulted in useless^18^ and wasteful,^19^ and even dangerous.^20^

Nevertheless, there is also another category of morally significant epistemic failure in this context: Framing of ethical choices as a simplified dilemma between mutually exclusive value options. Doing this can be superficially appealing, as it simplifies the problem and deceptively suggests a quick exit from the quandary. The infamous switch-case trolley problem with its numerous variations—often successful in teasing out moral intuitions from undergraduate ethics students—is a poor engineering model for decision making in real life. Applying ‘trolley thinking’ to the design of complex systems does not just ask the wrong questions, it also presents us with morally problematic choices. It may misrepresent the values at stake or misrepresent available options (such claiming that health surveillance requires us to either choose between privacy or health).^21^ Similarly, some technical tools presented as alternative to blanket lockdowns are framed in terms of a dilemmatic choice between privacy or a functioning economy.^22^ More subtle variations of such false dilemmas frame technological choices with claims that collected data can be either perfectly anonymous or useful for the intended epidemiological purpose.

These are classic instances of false dilemmas when evidence for the truth of a disjunctive premise is missing, or the disjunctive premise is evidently false. Such framing is particularly hazardous in the context of ‘emergency thinking’ when technological solutions are presented as constraints on our moral choices, rather than a path towards moral progress through innovation expanding our set of choices (van den Hoven et al. 2012). To avoid such technological determinism and to escape prearranged choices that lead to tragic moral dilemmas, we need to elucidate several myths that perpetuate this kind of a treacherous ‘state of exception’ logic.

### Myths of emergency

The prevalence of so-called ‘psychological disaster myths’ is well documented in disaster sociology and mass psychology (Tierney et al. 2006). This is a broad set of beliefs that, in emergency situations, members of the public are prone to panic, helplessness, and antisocial behaviour. These myths have been refuted by the empirical studies demonstrating that mutual support, coordination, and adaptive actions are often shown by those affected by disasters (Norris et al. 2008). This suggests that citizen participation is a fundamental element of community resilience. Accordingly, the effective engagement strategies to involve communities and prosocial virtues are crucial to the success of public health measures in the context of COVID-19 pandemic (Lau et al. 2020; WHO 2020).

Such collective resilience, however, can be undermined by the coercive top-down emergency response strategies. Based on the presumption of ‘disaster myths’ and a dysfunctional public, these responses restrict information and exclude affected members of the public from participating in their own protection, undermining a sense of agency and ability to cope (Drury et al. 2013). In the context of emergency many find themselves—as we have outlined above—in the grip of ‘moral overload’. Developers of technical solutions must facilitate problem solving in morally (over)loaded choice situations through the reduction of uncertainty and the proliferation of options that reduce the number and likelihood of tragic choices, instead of trapping users in the false dilemmas of choice between crucial activities and surrender of privacy.^23^

Solutions built on the myth of malicious and non-altruistic behaviour in disaster situations, not only perpetuate helplessness but also introduce harms of mass fear escalation, and the unacceptable stigmatisation of patients. This is especially dangerous as stigmatisation and victimisation of COVID-19 patients can be exaggerated by the assumptions (often probabilistic) regarding their infection status.^24^ This is particularly disturbing as we witness examples of public officials using the term ‘contact-tracing’ as a synonym for criminal investigation.^25^ Furthermore, we see that such problematic assumptions can become combined with opaque and inscrutable algorithmic governance tools used to impose restrictions on fundamental human rights.^26^

### Myths of privacy

Similarly to ‘disaster myths’, there are ‘myths of privacy’ that are well known to surveillance researchers. Privacy as a human right is too often misleadingly represented as simply an individual value. This is a false characterisation as privacy is more plausibly conceived as both an individual value and part of the common good, in the same way as health is both valuable for us as individuals and for society. Privacy is not reducible to mere psychological comfort—a myth often perpetuated by ad-tech companies. In fact, what is often presented as ‘acceptance’ of invasive surveillance by software users is the result of deliberate efforts to misguide and nudge users towards privacy-disclosing behaviour, exploiting numerous psychological biases (Acquisti et al. 2015).

Privacy harms are not reducible to feeling psychological discomfort; they carry real threats to human wellbeing and safety (van den Hoven 2008). In the current pandemic we have already witnessed examples of such harms ranging from online harassment,^27^ blackmail,^28^ phishing attacks,^29^ perpetuation of discrimination^30^ to physical aggression towards de-anonymised COVID-19 patients.^31^ The risks associated with attenuating privacy rights also introduce systemic social threats, and the distortion of social relations (Chaum 1985; Gasser et al. 2016). Unfortunately, collecting private data is far cheaper and technologically easier than effective anonymisation and other data protection measures, especially in the context of mobility data (Montjoye et al. 2013) and health data (Rocher et al. 2019). Any surveillance systems, including health ones, are prone to path dependencies of a technological and an institutional character.

Reverse engineering COVID-19 related apps has already shown extensive private data collection involving advertising, data analytics, and elevated permissions.^32^ The choice of certain solutions not only can open the door for malicious actors pursuing their interests, but can also ‘normalise’ the most dystopian scenarios. This threat is rapidly unfolding, with surveillance companies such as Palantir infiltrating critical national infrastructures,^33^ and major Chinese surveillance companies complicit in the oppression of Uighur Muslim minorities in China selling COVID-19 tracking tech worldwide.^34^

### Myths of big data and AI

Privacy myths often go hand in hand with ‘big data myths’. Together they perpetuate false beliefs that the expanding of the collection of private data always translates to increased value and added knowledge. The problem is not only that seeking ‘comprehensive data’ may be simply unattainable in the context of profound uncertainty surrounding novel pandemic, such focus also obscures crucial ethical concerns (Taylor 2020). These are closely related to ‘AI solutionism’, that is, the belief that feeding more private data into machine learning algorithms always provides new valuable insights, obfuscating questions on the moral appropriateness of such solutions.^35^ The misplaced concept of ‘new knowledge’, persistent in AI development can be attributed as the main culprit of ‘solutionism’. While machine-learning algorithms can discover previously undetected patterns in data sets, pattern discovery does not necessarily translate into generating new knowledge for the users of these tools. Discovered patterns can be scientifically insignificant, unsupported by empirical evidence, or simply irrelevant in the application context.

This stance is also characterised by the decidedly uncritical view on the predictive power of AI solutions, detached from the reality of application contexts. It often ignores such critical components as involvement of relevant domain expertise, quality of data sets, and limited universalisability and generalisability of data models. Unfortunately, it is common for AI developers to propose solutions that ignore one or more of these components. At some level, all data models are simplified representations of reality, more simple formal models, though, are generally more tractable for machine learning. This creates an incentive to abstract from the wider context of the problem for the sake of its tractability. The proposals on the use of AI facial recognition tools to for the diagnosis of COVID-19 are emblematic of these issues.^36^ The problem is not only that these (often vapourware) proposals, can introduce a false sense of health safety, but also that they solidify morally unacceptable business models based on the abuse of private data.

The second myth is that the private companies providing data analytics services are best able to manage this data. Profiting from this myth, some surveillance companies have tried to attain tenders for running public services, aiming to make themselves indispensable maintainers of critical infrastructures.^37^ This process is hazardous for at least two reasons. First, it risks undermining the integrity of public services, creating ‘moral fog’ (Cocking and Van den Hoven 2018) that can obscure our view of the role and function of spending taxes.^38^ Second, it undermines efforts against the normalisation of surveillance, and solidification of structural power asymmetries excluded from democratic oversight.

### Myths of data economy

The involvement of commercial companies whose primary business models are surveillance-based should give us grave concerns.^39^ Granted, initiatives to assist medical and government authorities can signal sincere efforts to help at this unprecedented time. However, one problem with repurposing commercial surveillance tools is that they may simply not be fit for purpose in a variety of ways. Simultaneously, legalising unethical and illegal practices (by the standards of EU's GDPR) amounts to ‘COVID-washing’, that is, the practice of dressing up nefarious business models as COVID-19 fighting initiatives.

These practices largely stem from and are defined by the shadowy world of private data markets. Here, marketing and advertising models based on direct targeting of consumers, cross channel tracking, and engagement metrics perpetuate a race between data collectors to collect as much data as possible. Skewed market incentives are further perpetuated by the prevalence of fraudulent traffic in online advertising, creating further incentives to collect even more private data for fraud mitigation (Pearce et al. 2014).^40^

A related myth—often used to justify commercial surveillance—is that increases in data collection lead to more equitable distributions of societal benefits. This, however, has never been shown to be the case. On the contrary, increased collection of big data and private data, in particular by corporations, has been shown to create persistent and ultimately unbridgeable power asymmetries. Rather than accruing societal benefit, ‘surveillance capitalism’ excels at leveraging information asymmetries for the benefits of concentrations of monopolistic power (Zuboff 2020). These models also have high margins of profit that are sustained through deliberately exploiting legal lag, often operating in the grey area of existing data protection regulations.

It is, therefore, misleading to assume that business models putting commercial interests of surveillance companies in direct contradiction with human rights, could be swiftly re-purposed for public health measures, even under such emergency conditions.^41^ And yet we observe intensified marketing campaigns—again smacking of ‘COVID-washing’—seemingly devised to brush aside these contradiction or avert public attention from them. Various companies, including malware producers^42^ and companies selling surveillance,^43^ are engaging in this mass rebranding of surveillance products.^44^ Non-consensual collection of private data from GPS and data points of mobile devices, smart city sensors, existing IoT deployments, mobility services, and advertising data silos are getting actively repainted as valuable public services.^45^ These practices have already been touted as necessary for the recovery of economy post crisis and returning to a ‘new normality’.^46^ Furthermore, this is a wider systemic issue that goes beyond privacy considerations. Even if privacy trade-offs are solved, this still creates enormous leverage for private companies that control crisis management infrastructures.^47^.

## Technological developments

### Automated contact tracing

Contact tracing is a tool for containing or slowing the spread of an infectious disease that has been used for many years by health care professionals (Klinkenberg et al. 2006). In its manual form, this method mostly relies on interviews to identify the potential contacts of a COVID-19 patient, in order to inform them of the measures they should take in order to prevent further transmission of a disease.^48^ The extreme approach to the digitalisation of this process is the aggregated use of all possible data sources, including GPS location data, cell phone location, travel data, and even surveillance cameras to recreate possible contacts of an infected patient (Zastrow 2020).

First, such sweeping data collection can hardly be reconciled with right to privacy given the opaque, and non-consensual character of the data collection, and how it is based on arbitrary criterion of proportionality. Second, any centralised repository of private data created for the purposes of contact tracing app presents a highly desirable target for cyber-criminals and has enormous potential for data abuse by trusted parties. Finally, the implementation of smartphone apps for contact tracing presents us with hard choices not only between specific architectures and security models, but also between assumptions about users’ behaviour. Choosing, for instance, to include the self-reporting of symptoms of users, rather than verified infected individuals, can cause cascading effects through the development cycle of these products.

All these issues create serious obstacles to the ethically justified implementation of contact tracing apps (Loi 2020). They also undermine public trust, hampering uptake of such apps, which is required if they are to be efficient. So far, there is strikingly little conclusive empirical evidence as to the efficacy of such apps (Braithwaite et al. 2020). Some suggest that an adoption rate by 60% of population might slow the rate of virus transmission,^49^ while others suggest that, even with the adoption rate above this percentage, they have limited effect.^50^ In terms of uptake, even the most successful app to date—the ‘Ranking-19’ app—used by nearly 40% of Icelandic population, has demonstrated negligible impact.^51^ Furthermore, difficulties in achieving sufficient levels of uptake around the world, caused by the lack of public trust, raises thorny questions about the use of such tools.^52^ Lack of balance between the promised benefits and privacy costs is perhaps their greatest weakness.^53^ Some, such as the original NHSX UK app, have now simply been abandoned by the government^54^; others have been scrapped because of the assessments of national data protection authorities.

At the time of writing (July 2020), automated contact tracing apps have branched into different directions. One is an approach that limits app functionality to exposure notification, based on a decentralised architecture. This side-steps some of the privacy and security pitfalls associated with centralised data collection. The second direction is the implementation of app based contact tracing (possibly combined with other surveillance tools) limited to workplaces or education institutions taking place in US, in which absence of relevant regulations can make these into de-facto mandatory requirements for employment.^55^ On top of this, in countries with little democratic oversight, COVID-19 ‘super apps’ seem to be rapidly evolving into permanent social control tools.^56^

The ‘exposure notification’ approach was initially proposed by the developers of Decentralized Privacy-Preserving Proximity Tracing (DP-3T).^57^ This was spearheaded by Apple and Google when these companies integrated a similar protocol in their mobile operating systems.^58^ The feature has been implemented as an application Programming Interface (API), which is only available to apps from healthcare authorities that have been vetted by Apple and Google. It is expected that this will be integrated at the operating system level at a later date. This approach does not aim to replace or emulate manual contact tracing; rather, it informs individuals about possible exposures to infection. It has been suggested that this can be achieved with the minimised collection of private data (only proximity to other phones is recorded without any location data) that uses pseudonymous, temporary identifiers.^59^

This approach works with smartphones that can broadcast random, temporary identifiers using a Bluetooth Low Energy (BLE) protocol. Additionally, each device using the app listens, records, and identifies other smartphones equipped with the app that came into close proximity. If an individual tests positive for COVID-19, these anonymous identifiers are published on a server (without letting it learn real identities) and any app equipped smartphone that records them notifies its owner about potential exposure. This approach can be considered decentralized insofar as the management of identities is implemented at the protocol level, and not dependent on a single trusted entity. No medical authority, nor any other centralized party, can infer the identities of users without the their explicit consent. Another clear advantage of this approach is that is does not create a centralised silo of personal data that could be abused by a trusted party or breached by an adversary. Some early findings from the deployment of such app in Switzerland could suggest certain effectiveness of this solution (Salathe et al. 2020).

This is not to suggest that this approach is problem free. It has already been claimed that existing surveillance systems collecting Bluetooth signals (such as scanners used in retail marketing)^60^ could be leveraged to de-anonymise users of this protocol.^61^ Notably, the efficacy of the Bluetooth signal for the assessment of infection risk has also not been resolved from an engineering point of view. While it does provide better accuracy than the GPS signal, Bluetooth does not accurately estimate distance due to various signal interferences (Leith and Farrell 2020b).^62^ It is also not clear whether apps based on this protocol will introduce further functionalities at a later stage, that are added on top of ‘exposure notification’. The latter is a crucial concern given that even seemingly minor design choices can profoundly affect privacy-vs-efficacy balance considerations.

Finally, even though implementation of identity management and data collection is decentralised, both Apple and Google act in this scheme as trusted parties. Any changes in protocol can be pushed onto users’ phones with future operating systems updates.^63^ It has been already reported that integration of Google Play services in the Android version of exposure notification protocol potentially allows fine-grained location tracking via IP address, and other identifiers by Google (Leith and Farrell 2020a). Given that the Apple and Google duopoly possesses the control over the smartphone market, they effectively would have the capacity to dictate the standards of COVID-19 containment measures to national governments around the world. The worry is that the prevention of function creep and dismantling of the system after the crisis becomes entirely dependent on what takes place in the corporate boardrooms of Apple and Google.

### AI and algorithmic governance

The deployment of Artificial intelligence (AI) tools in the context of the COVID-19 crisis, has been considered in various applications, ranging from medical research to optimising the availability of medical supplies. This means that immediate concerns relating to the risks to privacy or other human rights may have been not immediately apparent or may have been purposely ignored. At the same time, many of these tools should be viewed as problematic. Two acute areas of concern are: (1) appropriateness of implementing AI in these contexts; and (2) attempts to deploy tools that will be ultimately detrimental to social, political, or other forms of collective interest.

Appropriateness of implementations is contingent on domain-subject experts in the development and assessment of these tools. Some potentially promising applications, built in collaboration with medical researchers, include tools used to assist health care practitioners in diagnosing lung-scans of COVID-19 patients.^64^ These tools can be used to recognise patterns in lung tissue when applied to computer tomography scans.^65^ It is necessary, however, to ensure that early and experimental solutions are not presented as an immediate replacement of human expertise. In addition to this, we must ensure that the relevant medical or ethical safeguards are not side-stepped under the guise of emergency.

If such systems become deployed at scale, any mistakes in their design could cause cascading false-positive and false-negatives with tragic consequences. These worries are especially evident in the context of speculative applications, such as AI diagnosis of COVID-19 infection, based on the sound of the patient’s voice.^66^ Efficiency and appropriateness of these tools requires close scrutiny, particularly as AI solutions are increasingly proposed as decision-making tools in addition to diagnostic ones. Some of these speculative solutions are even being actively marketed, such as wearable devices claimed to provide early diagnosis through the collection and analysis sleep, heart rate, body temperature, and respiratory function data.^67^

Apart from questionable efficacy, these solutions raise the questions of extensive centralised data collection, as there is currently no viable AI-based analytics proposal (such as federated learning) in these contexts. Besides the issues of privacy and data abuse for commercial purposes, the opacity of data use highlights the risks of automated decision making and algorithmic governance.^68^ In some countries, algorithmic governance tools are already deployed under the pretext of emergency measures, eroding human rights and opening the floodgates for future technological social-control for political or economic means.^69^ Profound asymmetry between profiled individuals and entities deploying and controlling such systems leaves little space for any ethical justification in the support of these tools. One layer of this asymmetry stems from the input data obtained through the non-consensual surveillance of individuals. Another layer of asymmetry is the opaque ‘black-box’ nature of algorithmic assessment, arguably incompatible with the requirements of proportionality.^70^

AI based tools implemented as a mechanism of ‘algorithmic governance’ could, therefore, enable the future abuse of private data, arbitrary violation of human rights, and society wide mechanisms of intimidation. Opacity and asymmetry in the context of perceived emergency, create situation in which dangerous socio-technical systems become implemented without public scrutiny and proper impact assessment.^71^ Unsurprisingly, this is also seen as a window of opportunity by malicious actors, such as providers of malware and spyware, to legitimise their business models as socially acceptable through the practice of ‘COVID-washing’.

Finally, it is important to note that these risks are not limited to the threats posed by malicious actors. Deploying these tools creates market incentives for established technological companies^72^—and even academic researchers—to join the AI surveillance race.^73^ The lack of critical scrutiny and perceived epistemic authority of technological experts and researchers creates self-perpetuating cycles of development.

### Immunity passports

The initial idea of so-called ‘immunity passports’ emerged from the assumption that blood tests could identify antibodies produced by the immune system when it encounters SARS-CoV-2 virus. Since such antibodies are unique to particular viruses, their presence would indicate prior exposure to the virus and a sustained immune response to it. The hope was that such response might provide lasting immunity from the disease, therefore permitting people who have developed immunity safely return to work. At the time of writing (July 2020), studies of the mechanisms of immune responses to SARS-CoV-2 are inconclusive, so we cannot confirm that initial infection provides subsequent immunity to COVID-19 (Deeks et al. 2020). This suggests that while such tests could have an important public health role, at least in terms of mapping the transition of the disease, their value is questionable.^74^

Given the fallibility of current tests, the risk of false positives and false negatives is high. For this reason the WHO currently advises against implementing certification, until further evidence of sustained immune response becomes available.^75^ The lack of scientific evidence, however, has not stopped various national governments and companies from actively considering such ‘immunity certificates’ as an alternative to blanket lockdowns.^76^ It has also been argued that, in the absence of scientific evidence for the accuracy of antibody tests, such systems could be used to streamline exchange of other medical information such as results of negative tests for COVID-19.^77^ This idea has rapidly gained traction in digital identity systems, as a potential type of digital credential for the proof of vaccination (once a vaccine become available).^78^

Taken together, these developments could be considered within a general trend to medical certificates in digital form. While in some specific contexts such solutions might be desirable (data exchange between medical institutions, for example), attempts to introduce such digital medical certificates on a societal scale invoke grave moral concerns. The worry is that the ‘emergency context’ lends itself to fast-track scientifically questionable solutions, while side-stepping proper ethical evaluations.

Even in the hypothetical scenario where antibodies testing of vaccination could confer valid evidence of immunity, the very idea of ‘immunity certificates’ could be said to be ethically controversial. For one, if normalised, such practice may create skewed economic incentives for people to obtain immunity at the cost of contracting the virus. It also opens the door to discriminatory behaviour,^79^ both towards individuals without immunity, and individuals who may have had the infection. Benefits conveyed by such credentials may well introduce incentives for the black market trade in fake certificates.^80^

There is also a danger that such systems might be implemented or co-opted by the companies operating commercial surveillance infrastructures, based on a centralised systems and aggregated identities, such as proposals on “coronavirus-immunity registry.”^81^ We should also be wary that a crisis can obscure developments of previously rejected national ID schemes with opaque purposes under the guise of ‘COVID-washing’.^82^

Proposals for the alternative decentralised architectures for identity management solutions based on Self-Sovereign Identity (SSI) systems, however, are not free from ethical apprehension either.^83^ The appeal of such systems lies in their capacity for data-minimised presentation, and the sharing of medical credentials between individuals and different medical organisations, providing interoperability of identification standards, and verification of authenticity. The key worry here is the lack of maturity of SSI standards and blockchain-based infrastructures, used for the implementation of such systems.^84^ Other open issues for such systems, include mechanisms for the onboarding of data and non-transferability of credentials. Moreover, there is a fundamental worry that, just like other promising cryptographic solutions (Rogaway 2015), ‘SSI’ could be co-opted into a speculative marketing label, and be used to disguise ethically problematic schemes.^85^

Regardless of the chosen technical architectures, any solutions for digital medical certificates for COVID-19 will have to pass the test of efficiency, proportionality, and ethical acceptability. The latter requires not only valid scientific basis, but context-specific ethical frameworks for the assessment of these solutions, developed with the participation of all affected stakeholders. Otherwise, driven by commercial or malicious interests, such solutions may become a permanent fixture of systematic discrimination and bio-surveillance.

## The path forward

As we have seen, development of information technology tools capable of aiding the fight against COVID-19 has quickly generated a vast volume of innovations. Can these innovations form the basis of responsible policy interventions? Can we develop these technologies in a way that is ethical as well as effective? In the following three sections, we show how these questions can be answered from research in *Responsible Innovation* and *Ethics by Design*. Just as the short life-cycles of *Drosophila*—fruit flies—provide an indispensable research tool for geneticists, accelerated innovation cycles of contact tracing apps, and the other solutions we have outlined, provide invaluable insights on the philosophy and ethics of innovation. Furthermore, the crisis has acutely demonstrated that we not only need to scrutinise the trajectories of technological developments, but we also must propose new models of resilient techno-social systems. To make these systems more resistant to future shocks with the help of digital solutions that enhance flexibility, coordination, and knowledge sharing.^86^

We believe that there are three vital lessons that can be learned from the ethics of information technology that are especially relevant to dealing with COVID-19. First, if we take our shared values seriously, then we must design for them and shape new technology in accordance with them (Design for Values). Second, in proposing innovations to solve urgent societal problems, we have to proceed responsibly and strive to fulfil as many of our obligations as is feasible (Responsible Innovation). Finally, we need to cast our net wide. This means that we must include the greatest possible variety of disciplines and stakeholders. Solutions need to be subsumed in a sufficiently generous systems perspective, without which we will be unable to see the interactions between complex systems (Comprehensive Engineering).

### Design for values

The recent surge of technological solutions to the COVID-19 pandemic should remind us of the fact that technology does not only (and does not always) deliver its promised functionality. Certain technologies, architectures, applications, or services may promote the ideals, conceptions of society, or preferred socio-economic models, of the designers and developers whether this is done explicitly or surreptitiously, whether this is intended or not (van den Hoven et al. 2015).

COVID-19 reminds us that our thinking and decision-making in crisis and emergency mode, under conditions of deep uncertainty and incomplete information, only adds to the risk of obscuring the important *value laden aspect* of technology.^87^ This may not only lead to a distorted and flawed understanding of the values at play in large-scale experiments with high-risk technologies such as AI, but may also lead us to miss better options. Furthermore, it may cause us to betray public acceptance, therefore undermining trust in politicians and public health institutions.^88^

This means that the crisis context, along with the high stakes of rushed technological choices, makes it especially important that particular values are made explicit. It also means that technological implementations are carefully scrutinised and meticulously evaluated in practice. These concerns cannot be neglected when we witness disturbing developments in COVID-related technologies such as surveillance wearables^89^ and digital immunity passports.^90^

It is also clear that mere declarations of value commitments in this context are not sufficient, as is evident in the deployment of hastily implemented ‘privacy preserving’ contact tracing apps with clear security flaws.^91^ We need to tend to the coherence of our assumptions, expectations, predictions and beliefs, test the practical consistency of our moral and political judgements and evaluations, and systematically and transparently translate our shared values into design principles and technological requirements.

The methods of value sensitive design explicitly support reflection on ethical considerations and moral values at early stages in the development of technology, especially in terms of design and research (van den Hoven et al. 2017). This ensures that ethics and discussions about relevant values are not separated from what we actually do when fighting the crisis, or from the socio-economic consequences of our choices. This prevents derailment of our intentions, for instance by turning legally voluntary solutions into obligatory ones.^92^ It ensures that our values are effectively applied, i.e. ‘functionally decomposed’ and operationalised, in the same way that other high level and abstract requirements are dealt with in engineering and design work.

### Responsible innovation

Successfully implementing innovations that are necessary to deal with intelligent (and possibly intermittent unlocking) scenarios, requires appreciation of value conflicts and trade-offs that present themselves in the process. Value-sensitive design aims to go beyond mere declarations of value commitments and see moral values as non-functional requirements for which we ought to design, transparently, systematically and demonstrably.

We must, in particular, avoid falling into the trap of false moral dilemmas and tragic choices dictated by technological determinism, market failures, and private interests. We see all these factors in action in the rapid installation of commercial surveillance infrastructures that have been marketed as the only solution to the crisis.^93^ The main oppositions between health and the economy, between the economy and privacy, between privacy and accountability should not be accepted at face value.^94^ They could prove to represent genuine dilemmas, but often, there are third options that go unmentioned or are not explored on conceived. Responsible innovation typically tries to transcend the dilemmatic character of these oppositions and encourages us to think of smart solutions, so we can avoid making tragic choice.^95^

Taken as an activity or process, responsible innovation enables moral agents to obtain relevant knowledge on the consequences of their actions, as well as evaluating them effectively in terms of relevant moral values. Responsible innovation, therefore, differs from approaches to innovation that are concerned with simply adding new functionality, as it attempts to aim at solutions to significant social problems by adding morally relevant functionality (van den Hoven et al. 2014; von Schomberg and Hankins 2019). This creates new ‘third choices’ beyond binary dilemmas, leading to morally improved situations in which we can do more good than was previously possible.

### Comprehensive engineering

By overly singling out a single subset of socio-technical systems—such as contact-tracing apps as a ‘silver bullet’ solution, for example—we risk ignoring the wider systemic view. We also miss how the success of these apps also depends on the availability of medical and other infrastructures.^96^

Comprehensive engineering, then, is the third key component in thinking about and dealing with innovations that can aid engineers, developers, and providers of information technologies to responsibly respond to the global challenge of the current crisis.^97^ Adequate solutions to systemic problems—especially a pandemic—are always *systems solutions*, which take into account many technological aspects, human behaviour, values, and norms. Comprehensive engineering is a form of complex systems (dynamics) engineering (complex adaptive systems) accommodating different aspects of socio-technical systems: systems dynamics and complexity, moral, social (legal, institutional, behavioural and economic, cultural) and technical aspects.^98^ This is an interdisciplinary and multi-disciplinary approach to engineering, offering comprehensive analyses and future solutions.

To ensure the fair distribution of risks, benefits, and responsibilities, decision makers need to be able to think comprehensively about systems in a sufficiently rich way. The challenges of the current crisis make it obvious that ignoring even a single component in a system—and how it is dynamically related to other parts—can undermine the rest of it.^99^ Thinking back to contact-tracing apps illustrates this. Here we can see how far some of the proposed solutions that deliberately focus on specific system aspects such as convenience of data aggregation, may fail to achieve public trust and the sufficiently wide adoption prerequisite to their efficacy.^100^ Comprehensive engineering is not an approach that leverages understanding of social components to achieve successful deployment of technical systems. Rather, it takes a holistic view of the ‘systems of systems’ comprised of moral reason, institutions, incentive structures, and marker orderings, procedures, and individual humans with their own mental states who act in these contexts (Van den Hoven 2019).

Predictive models, contact tracing tools, COVID-19 testing policies, social and physical distancing practices, compensation schemes for SMEs, nationalisations of essential industries, online learning and distance education, policing and enforcement strategies, public perception of health authorities and government are interrelated—they must be viewed as such. If we orientate our pandemic strategies by viewing these components separately they will certainly fail. Comprehensive engineering of responsible digital solutions tries to understand how constraints and affordances of normative, social and institutional structures interact with technical components, technical processes, and technical infrastructures.

## Early initiatives

We finish by noting that there are already several promising initiatives from key players at the forefront of the fight against the pandemic. Our list of these initiatives is not intended to be exhaustive; rather they have been chosen as representative examples of excellent practice. Each of these documents highlight the very real dangers of technological and policy solutions to the pandemic that have not received the requisite ethical oversight. It is our hope that these early initiatives will have some effect on the initial deployment of emerging technologies in the fight against COVID-19.

First, the EGE’s (European Group on Ethics in Science and New Technologies) ‘Statement on European Solidarity and the Protection of Fundamental Rights in the COVID-19 Pandemic’ lays out a distinctively European approach to the values and principles that should govern responses to the crisis by individual member states and by the European Union itself. The EGE proposes that Europe should lead in terms of a quintessentially ethical response to the crisis, one that safeguards and promotes the values of solidarity, trust and transparency, and human rights (2020, pp. 1–2). These values will be jeopardised by the privileging of economic concerns, so the EGE warns against market interference, the neglect of vulnerable groups (the elderly, single parents, at-risk children), and the formation of an ethically impoverished ‘new normal’ once the formal state of emergency has ended. To prevent this, the EGE calls for renewed attention to material support of these vulnerable groups, to decent funding of furlough schemes, and cross-national healthcare initiatives (2020, pp. 3–4). By showing wisdom and leadership during the pandemic, the Group suggests, the European Union will be able to formulate a viable strategy that shows how similar crises can be dealt with effectively in the future.

Secondly, the European Commission’s Group of Chief Scientific Advisors (GCSA) has issued a statement together with the EGE in conjunction with Peter Piot, world-renowned epidemiologist and special advisor to the President of the European Commission on the topic of giving of scientific advice to European policy makers during the COVID-19 pandemic. The statement highlights key issues regarding the use of scientific advice when creating policy directives to deal with pandemics. The authors suggest that the ‘complexity of the COVID-19 pandemic and its aftermath means that a multidisciplinary approach is required to develop advice’ (2020, p. 3). Given that our knowledge of a COVID-19 pandemic is invariably ‘uncertain and tentative’, they continue, it is essential that advice should be effectively communicated to policy makers and to the general public (2020, p. 3). Only when the scientific advice given by official advisors is open and transparent, and is based on the highest quality of evidence, can public trust be achieved. Finally, clarity on the governance arrangements and responsibilities in the networks—from science advisors to political leadership to medical agencies—is a critical requirement. The document concludes that, while COVID-19 pandemic presents an immense global challenge, it is one that can be prepared for in advance, with broad scientific consultation, analysis, and planning.

Third, SoBigData, a research initiative of the European Union's Horizon 2020 programme (Grant No. 654024), warns against a ‘’centralised approach’ to data collection and ‘location trackingtechnology’ (2020, p. 2). Their statement, titled ‘Give More Data, Awareness, and Control to Individual Citizens’, highlights the advantages of a decentralised approach to data collection. Prima facie this approach bears similarities to the recent Google/Apple collaboration (Sect. 2.1 of this Introduction), but it is distinctive in several ways. First, SoBigData proposes that each contact-tracing app user be granted exclusive control over their information, that this data cannot be shared without consent, and that sharing data is subject to strict individual oversight (2020, p. 5). Second, gathering data must be bound by limits of applicability and limits of lifespan (2020, pp. 5–6). This means that only data explicitly relating to COVID-19 can be shared, and that this must be destroyed once the pandemic has passed (these two stipulations frequently appear in the appendix of manifestos; see below). Finally, the data gathered should be of direct benefit to the individual user. For example, data should enable the user to modify their behaviour in ways that reduce the risk of exposure to SARS-CoV-2 (2020: 6). In sum, with adequate safeguards, SoBigData supports the use of contact-tracing technology in containing the virus because it will ‘shorten the emergency period’ and direct medical resources (PPE, medicines, nurses, etc.) to regions that require them (2020, p. 2).

Fourth, Amnesty’s statement, titled ‘States Use of Digital Surveillance Technologies to Fight Pandemic Must Respect Human Rights’, stresses the dangers of tracing apps for human rights and values. Data-driven technologies require a bespoke ethical approach to the safeguarding of human rights, it argues, and the statement highlights eight key areas of concern. These include ensuring that the gathering of data is ‘lawful, necessary and proportionate’, so that using apps to fight the virus does not become an ‘excuse for indiscriminate mass surveillance’ (2020, p. 1). Mass surveillance can be guarded against by ensuring that the collection of data is (1) ‘timebound’, that (2) it only relates to the ‘purposes’ of dealing with the COVID-19 pandemic, that (3) they are ‘secure, and that they are strictly anonymised (2020, pp. 1–2). In addition to this, Amnesty strenuously warns against sharing gathered data with third parties (companies or commercial interests, say), and recommends that the gathering of data should be located outside the purview of security or intelligence agencies. These checks and balances aim to ensure that the data we gather to fight COVID-19 is not enlisted for discriminatory purposes, especially against currently marginalised populations. There is a very real risk that the pandemic could entrench existing divisions.

Fifth, ICT4Peace’s statement, titled ‘Corona Pan(dem)ic: The Gateway to Global Surveillance?,’ also focuses on the challenges to human rights that COVID-19 tracing apps create. ICT4Peace point to a potential large-scale erosion of privacy if contact-tracing apps are not introduced with strict and binding ethical safeguards. While the World Health Organisation promotes contact tracing (both online and offline) in principle, the author notes, these services present a range of problems that do not beset traditional methods. Electronic contact tracing technologies have been adopted by a growing number of governments (twenty, at the time of writing), and can even be integrated with other surveillance technologies (heat sensors, surveillance drones, CCTV networks, etc.). The author also notes that the pandemic has affected the public flow of information. Repressive governments have used the events of early 2020 to curb information. Furthermore, misinformation on the causes and relief of symptoms is on the rise in Western democracies. Similarly to Amnesty’s analysis, ICT4Peace stresses that emergency measures must be ‘necessary’, ‘proportionate’and ‘time-bound’. Unless these factors inform our design of tracing technology, the authors caution, we may survive the medical effects of the pandemic, but the post-COVID-19 world may ‘violate human rights that protect [the] seed of humanity each of us carries within’ (2020, p. 7).

Sixth, the statement of purpose for The Confederation of Laboratories for Artificial Intelligence (CLAIRE) outlines the recent research activities of this group. CLAIRE is a ‘bottom-up, expert-driven, non-profit endeavour’, and its statement is a testament to the effectiveness of this mode of collective organisation, especially in a crisis scenario. The document outlines seven research foci to which CLARIE researchers have contributed since March 2020: (1) epidemiological modelling; (2) mobility and monitoring data analysis; (3) bioinformatics; (4) image analysis; (5) social dynamics; (6) robotics; (7) resource management (2020, pp. 3–7). In each of these areas, CLAIRE researchers have used their expertise to show how emerging technologies have capacities and affordances to improve our ability to respond to COVID-19. The teams of volunteer experts that have worked on these areas have identified ways that current research projects can be enlisted into this fight. This provides us with a useful overview of how European institutions have collaborated in response to the pandemic. In addition to this, as the Task Force Coordinators warn, it is ‘more than likely that our societies will be confronted in the not-so-far future with other crises of similar scale’ (2020, p. 7), so the second half of the document sketches a set of future recommendations. These include legislative changes that would facilitate the flow of information (aiding collaboration), the development of an European framework to openly manage medical data, and better collaborative networks between AI researchers and frontline medical professionals. The authors end cautiously, noting that ‘technologically easy to put in place systems that might be difficult to dial back once the crisis is over’, and warning that ‘we must develop standards and frameworks that permit rapid progress without eroding human dignity’ (2020, p. 11).

Finally, the World Health Organisation’s ‘Ethical Considerations to Guide the Use of Digital Proximity Tracking Technologies for COVID-19 Contact Tracing’lays out a comprehensive roadmap to the ethical development of contact tracing technology. This document was developed in consultation with a multi-disciplinary group of global experts, including the editor-in-chief of *Ethics and Information Technology*, Jeroen van den Hoven. The document begins by cautioning against blue sky thinking in this area. The authors note that currently there are ‘no established methods for assessing the effectiveness of digital proximity tracking’ (2020, p. 2). This means that these technologies ‘must be subject to rigorous review’, so that we can ensure that the ‘trade-off of privacy is proportional to the public health impact achieved’ (2020, p. 2). Compared to all the statements we include, the multiple authors of this document identify the largest number of ethical principles that ought to be taken into account in the development of COVID-19 track-and-trace technology. These include some of the ethical principles mentioned by the other included documents (sunset clauses, voluntariness, security, etc.), but the WHO’s statement also emphasises the importance of ‘independent oversight’, ‘participation of the relevant stakeholders’, ‘accountability protections’ (2020, p. 5). These additions emphasise that implementing contact tracing technology should be primarily viewed as a collective undertaking. It is not only a public health measure; it is a technology that can only work if we all regard ourselves (and are treated) as connected stakeholders. Doing this requires that contact tracing apps are initially designed to respect user’s rights, that they inform users about how their data will be used (and allow them to prevent future changes), and make app developers (or their public-sector customers) fully accountable for their products.

These manifestos and organisational statements are reprinted with permission, and are added as an appendix to this special issue. It is our hope that they provide the reader with a glimmer of hope while appraising the quick-response articles on the ethical challenges that the COVID-19 pandemic presents. Each of the submissions to this special issues, confronts existing initiatives with a range of important topics to consider if we are to employ emerging technologies in the fight against COVID-19 in a way that is ethical, fair, and just. We hope that presenting these articles together with details of the existing initiatives goes some way to illuminating the ethical uncertainty by which we are currently surrounded.

## Footnotes

1 This editorial essay was originally drafted in the summer of 2020 and wrapped up in autumn of 2020. Nevertheless, due to the publication backlogs with the special issue of “Ethics of Information Technology in the COVID-19 Crisis”, this editorial is coming to publication in the Spring 2021. Looking back, we can consider it as a snapshot in time, when certain technological solutions were at the various points in their ‘hype’ and development cycles. At this point in the pandemic, some of the moral risks (such as segregation between vaccinated and unvaccinated individuals) seemed remote and far-fetched. Fast forward several months, and we find ourselves in a significantly changed technological landscape. This shows that ethical assessment of emerging technologies is no longer an exercise in reflection so dear to traditional philosophy, but is fast-paced research following dramatically shortened lifecycles of technological solutions. Ethicists of technology are becoming less armchair philosophers, and more akin to a scientist studying drosophila flies. Contact-tracing applications are no longer touted as the most promising technological alternative to blanket lockdowns. It is not fair to say that the usefulness of contact-tracing applications has proved to be lacking, rather they are getting more sober assessment as but one of the tools available to us in the fight against COVID-19. At the same time, digital medical certificates are rising at the top of the public spotlight with overly-enthusiastic proposals on digital ‘vaccine passports’. These solutions raise different ethical issues, but a number of apprehensions persist: Which entities will control such infrastructure? How will these infrastructures be integrated with existing medical information systems? How can we ensure that these technologies do not become a permanent tool of social access-control? More disturbingly, it seems that pitfalls and shortcomings of the first wave of contact-tracing tools have not generated sufficient awareness towards the complexity of creating a whole new ecosystem of health-surveillance tools. Any such system introduces new levers of control, new vulnerabilities, new attack surfaces for malicious entities. In that sense, we are not even past the risks of the first generation of contact-tracing apps, as these may easily become mules for surveillance and access-control ‘super-apps’, under the guise of health applications. The roll out of vaccines contributes to the cautious optimism, shifting the attention to different solutions. Extraordinary progress in the scientific understanding of COVID-19, new ways of treatment and prevention, show light at the end of the tunnel, giving hopes that humanity will overcome this cataclysm. Just as we should not relax diligence and caution prematurely, and we should continue to observe necessary hygiene measures, we should also heighten our awareness of moral hazards posed by the complex, large scale infrastructures for health surveillance. We stand dangerously close to the Rubicon of a new world in which the lines between the spheres of justice regarding sensitive private data collection and use are permanently blurred.

2 Privacy International’s Tracking the Global Response to COVID-19. https://privacyinternational.org/examples/tracking-global-response-covid-19.

3 noyb/GDPRHub. Data Protection under SARS-CoV-2 https://gdprhub.eu/index.php?title=Data_Protection_under_SARS-CoV-2.

4 ‘Coronavirus Monitoring Bracelets Flood the Market, Ready to Snitch on People Who Don’t Distance’ https://theintercept.com/2020/05/25/coronavirus-tracking-bracelets-monitors-surveillance-supercom/?utm_medium=email&utm_source=The%20Intercept%20Newsletter.

5 Facial Recognition Firms Pitch COVID-19 ‘Immunity Passports’ For America And Britain https://www.forbes.com/sites/thomasbrewster/2020/05/20/facial-recognition-firms-pitch-covid-19-immunity-passports-for-america-and-britain/.

6 China’s Virus Apps May Outlast the Outbreak, Stirring Privacy Fears https://www.nytimes.com/2020/05/26/technology/china-coronavirus-surveillance.html.

7 Europe Outbreak in Check But Virus Apps Struggle for Traction https://www.bloomberg.com/news/articles/2020-06-25/europe-outbreak-in-check-but-virus-apps-struggle-for-traction.

8 The rise and fall of Hancock’s homegrown tracing app https://www.ft.com/content/9446192a-aff1-4e95-93fb-a5adfbc7bbd5.

9 Major Security Flaws Found in South Korea Quarantine App https://www.nytimes.com/2020/07/21/technology/korea-coronavirus-app-security.html.

10 Norway and Lithuania have recently suspended their contact-tracing app due to privacy concerns https://www.theguardian.com/world/2020/jun/15/norway-suspends-virus-tracing-app-due-to-privacy-concerns. See also: https://globaldatareview.com/coronavirus/lithuanian-contact-tracing-app-suspended. See also: https://www.reuters.com/article/us-health-coronavirus-europe-tech/germany-flips-on-smartphone-contact-tracing-backs-apple-and-google-idUSKCN22807J.

11 In Response to Technical and Adoption Issues With TraceTogether App, Singapore Makes a Second Effort With an Always-Offline Contact Tracing Wearable https://www.cpomagazine.com/data-privacy/in-response-to-technical-and-adoption-issues-with-tracetogether-app-singapore-makes-a-second-effort-with-an-always-offline-contact-tracing-wearable/.

12 A new system uses software to dictate quarantines — and appears to send personal data to police https://www.nytimes.com/2020/03/01/business/china-coronavirus-surveillance.html.

13 Coronavirus opens door to company surveillance of workers https://www.politico.com/news/2020/06/26/workplace-apps-tracking-coronavirus-could-test-privacy-boundaries-340525. See also: Coronavirus: How much does your boss need to know about you? https://www.bbc.com/news/business-53109207.

14 Fearing coronavirus, a Michigan college is tracking its students with a flawed app https://techcrunch.com/2020/08/19/coronavirus-albion-security-flaws-app/?guccounter=1.

15 South Korea Holds Onto Patient Data From Prior Coronavirus, Worrying Privacy Groups https://www.npr.org/sections/coronavirus-live-updates/2020/06/30/884580723/south-korea-holds-onto-patient-data-from-prior-coronavirus-worrying-privacy-grou?t=1593683804978.

16 Can a Smart Watch Detect CovidCOVID-19? https://gizmodo.com/can-a-smart-watch-detect-covid-19-1833409102.

17 Underfunded Russian hospitals emerge as key vector for virus https://www.bloomberg.com/news/articles/2020-04-20/russia-s-underfunded-hospitals-emerge-as-key-vector-for-virus.

18 Nearly 40% of Icelanders are using a COVID app—and it hasn’t helped much https://www.technologyreview.com/2020/05/11/1001541/iceland-rakning-c19-covid-contact-tracing/.

19 Having spent £12 m on development, the UK now has no contact tracing app https://www.ft.com/content/9446192a-aff1-4e95-93fb-a5adfbc7bbd5.

20 These Chinese firms were blacklisted for Uighur oppression. Now they want to sell COVID-19 surveillance tools to the West. https://www.businessinsider.nl/blacklisted-chinese-firms-uighur-oppression-covid-19-surveillance-tech-2020-6?international=true&r=US.

21 Cellphone tracking could help stem the spread of coronavirus. Is privacy the price? https://www.sciencemag.org/news/2020/03/cellphone-tracking-could-help-stem-spread-coronavirus-privacy-price.

22 Your Boss May Soon Track You At Work For Coronavirus Safety https://www.npr.org/2020/05/08/852896051/your-boss-may-soon-track-you-at-work-for-coronavirus-safety?t=1589005431904.

23 Brown University is using a new app from Alphabet’s Verily to bring teachers back to campus. Participants will have to consent to let Verily collect their data. https://www.fastcompany.com/90518685/brown-is-using-a-new-app-from-alphabets-verily-to-bring-teachers-back-to-campus.

24 Don't Criminalize The Coronavirus https://www.wbur.org/cognoscenti/2020/04/16/police-coronavirus-ivan-espinoza-madrigal.

25 Mass gatherings, erosion of trust upend coronavirus control https://apnews.com/88cc916ad9611fed045001dcd5010c2f.

26 Chinas Code-System: Wie die Coronakrise zu noch mehr Überwachung führte https://www.handelsblatt.com/technik/digitale-revolution/digitale-revolution-chinas-code-system-wie-die-coronakrise-zu-noch-mehr-ueberwachung-fuehrte/25653166.html?ticket=ST-2217109-cHDGsmYkcohxcB6CaVkP-ap1.

27 In Russia, Coronavirus Patients Fight Infection, Stigma and Harassment https://www.themoscowtimes.com/2020/04/15/in-russia-coronavirus-patients-fight-infection-stigma-and-harassment-a69993.

28 'More scary than coronavirus': South Korea's health alerts expose private lives https://www.theguardian.com/world/2020/mar/06/more-scary-than-coronavirus-south-koreas-health-alerts-expose-private-lives.

29 NHS contact tracing undermined by hackers sending fraudulent warnings to public https://www.telegraph.co.uk/news/2020/05/30/nhs-contact-tracing-undermined-hackers-sending-fraudulent-warnings/.

30 South Korea struggles to contain new outbreak amid anti-gay backlash https://www.theguardian.com/world/2020/may/11/south-korea-struggles-to-contain-new-outbreak-amid-anti-lgbt-backlash.

31 Stoning of residence of family being ravaged by COVID-19 condemned https://news.mb.com.ph/2020/04/05/stoning-of-residence-of-family-being-ravaged-by--19-condemned/.

32 ‘Defensive Lab Agency’ actively tracks new Android applications that are published in response to COVID-19 and analyses them for security and privacy. https://forensic.defensive-lab.agency/covid/.

33 Under pressure, UK government releases NHS COVID data deals with big tech https://www.opendemocracy.net/en/under-pressure-uk-government-releases-nhs-covid-data-deals-big-tech/.

34 These Chinese firms were blacklisted for Uighur oppression. Now they want to sell COVID-19 surveillance tools to the West. https://www.businessinsider.nl/blacklisted-chinese-firms-uighur-oppression-covid-19-surveillance-tech-2020-6?international=true&r=US.

35 UK government using confidential patient data in coronavirus response https://www.theguardian.com/world/2020/apr/12/uk-government-using-confidential-patient-data-in-coronavirus-response.

36 Faked Coronavirus Fever Detection, Athena Used Hikvision https://ipvm.com/reports/faked-corona?code=allow.

37 FEMA Tells States to Hand Public Health Data Over to Palantir https://www.thedailybeast.com/fema-tells-states-to-hand-public-health-data-over-to-palantir.

38 Vote Leave AI firm wins seven government contracts in 18 months https://www.theguardian.com/world/2020/may/04/vote-leave-ai-firm-wins-seven-government-contracts-in-18-months.

39 How big tech plans to profit from the pandemic https://www.theguardian.com/news/2020/may/13/naomi-klein-how-big-tech-plans-to-profit-from-coronavirus-pandemic.

40 There’s a ticking time bomb inside the online advertising market https://fortune.com/2015/07/01/online-advertising-fraud/.

41 Umstrittener Daten-Deal: Hessen setzt auf Palantir im Kampf gegen Corona https://www.heise.de/newsticker/meldung/Umstrittener-Daten-Deal-Hessen-setzt-auf-Palantir-im-Kampf-gegen-Corona-4707941.html.

42 FBI probes use of Israeli firm's spyware in personal and government hacks—sources https://www.reuters.com/article/us-usa-cyber-nso-exclusive-idUSKBN1ZT38B.

43 A US Senator Wants To Know Which Federal Authorities Are Using Clearview AI To Track The Coronavirus https://www.buzzfeednews.com/article/carolinehaskins1/senator-markey-clearview-ai-covid-contact-tracing.

44 Special Report: Cyber-intel firms pitch governments on spy tools to trace coronavirus https://www.reuters.com/article/us-health-coronavirus-spy-specialreport-idUSKCN22A2G1.

45 How mobility data could help governments track lockdown compliance https://venturebeat.com/2020/05/01/how-mobility-data-could-help-governments-track-lockdown-compliance/.

46 Coronavirus UK: health passports 'possible in months’ https://www.theguardian.com/politics/2020/may/03/coronavirus-health-passports-for-uk-possible-in-months.

47 When Google and Apple get privacy right, is there still something wrong? https://medium.com/@TamarSharon/when-google-and-apple-get-privacy-right-is-there-still-something-wrong-a7be4166c295

48 How Germany’s Relentless Contact Tracers Helped Beat the Virus https://www.bloomberg.com/news/articles/2020-05-18/german-success-in-contact-tracing-guides-new-jersey-and-new-york?srnd=premium-europe.

49 Digital contact tracing can slow or even stop coronavirus transmission and ease us out of lockdown. https://www.bdi.ox.ac.uk/news/digital-contact-tracing-can-slow-or-even-stop-coronavirus-transmission-and-ease-us-out-of-lockdown.

50 Simulation model shows that by themselves these apps have none or minimal effect on the spread of the virus. https://simassocc.org/assocc-agent-based-social-simulation-of-the-coronavirus-crisis/news-and-publications/.

51 Nearly 40% of Icelanders are using a COVID app—and it hasn’t helped much https://www.technologyreview.com/2020/05/11/1001541/iceland-rakning-c19-covid-contact-tracing/.

52 French virus tracing app flops with only 14 alerts https://medicalxpress.com/news/2020-06-french-virus-app-flops.html.

53 Surge of cases in Australia as government admits tracing app has not found any new contacts https://www.telegraph.co.uk/news/2020/06/29/surge-cases-australia-government-admits-tracing-app-has-not/.

54 The rise and fall of Hancock’s homegrown tracing app https://www.ft.com/content/9446192a-aff1-4e95-93fb-a5adfbc7bbd5.

55 Coronavirus opens door to company surveillance of workers https://www.politico.com/news/2020/06/26/workplace-apps-tracking-coronavirus-could-test-privacy-boundaries-340525.

56 China’s Virus Apps May Outlast the Outbreak, Stirring Privacy Fears https://www.nytimes.com/2020/05/26/technology/china-coronavirus-surveillance.html.

57 DP3T: Decentralized Privacy-Preserving Proximity Tracing https://github.com/DP-3T/documents.

58 Privacy-Preserving Contact Tracing https://www.apple.com/covid19/contacttracing/.

59 DP3T: Decentralized Privacy-Preserving Proximity Tracing https://github.com/DP-3T/documents.

60 Privacy trade-offs in retail tracking https://www.ftc.gov/news-events/blogs/techftc/2015/04/privacy-trade-offs-retail-tracking.

61 How Apple And Google Are Going To Enable Contact Tracing https://joekent.nyc/google-apple-contact-tracing.

62 See also: Inferring distance from Bluetooth signal strength https://medium.com/personaldata-io/inferring-distance-from-bluetooth-signal-strength-a-deep-dive-fe7badc2bb6d.

63 How Google Plans to Push Its Coronavirus Tracing Feature to Android Phones https://www.vice.com/en_us/article/dygbmj/how-google-coronavirus-contact-tracing-feature-update.

64 AI researchers support efforts to combat COVID19 https://covid19.claire-ai.org/. Imaging COVID-19 AI initiative is a multicenter European project to enhance computed tomography (CT) in the diagnosis of COVID-19 by using artificial intelligence. https://imagingcovid19ai.eu/.

65 Can AI diagnose COVID-19 on CT scans https://thehealthcareblog.com/blog/2020/03/23/can-ai-diagnose-covid-19-on-ct-scans-can-humans/.

66 The project, Corona Voice Detect https://voca.ai/corona-virus/. By voice or location, Israeli apps can determine your risk of coronavirus https://www.timesofisrael.com/by-voice-or-location-israeli-apps-can-determine-your-risk-of-coronavirus/.

67 Hype and hope: Wearables in the COVID-19 era https://www.engadget.com/hype-and-hope-wearables-in-the-covid-era-190006602.html

68 Amazon Touts AI for Social Distancing Amid Worker Complaints https://www.wired.com/story/amazon-touts-ai-social-distancing-worker-complaints/.

69 Chinese city plans to turn coronavirus app into permanent health tracker https://www.theguardian.com/world/2020/may/26/chinese-city-plans-to-turn-coronavirus-app-into-permanent-health-tracker.

70 Google tracked his bike ride past a burglarized home. That made him a suspect. https://www.nbcnews.com/news/us-news/google-tracked-his-bike-ride-past-burglarized-home-made-him-n1151761.

71 Palantir’s New ‘Driving Thrust’: Predicting Coronavirus Outbreaks https://www.bloomberg.com/news/articles/2020-04-02/coronavirus-news-palantir-gives-away-data-mining-tools.

72 Amazon will also use machine-learning software to monitor building cameras and determine whether employees are staying at safe distances during their shifts. https://www.reuters.com/article/us-health-coronavirus-amazon-com-masks-e/exclusive-amazon-to-deploy-masks-and-temperature-checks-for-workers-by-next-week-idUSKBN21K1Y6.

73 MIT uses wireless signals and AI to monitor COVID-19 patients at home https://www.engadget.com/mit-csail-coronavirus-patient-monitoring-device-190037775.html?_guc_consent_skip=1587902786. Stanford researchers propose AI in-home system that can monitor for coronavirus symptoms https://venturebeat.com/2020/04/06/stanford-researchers-propose-ai-in-home-system-that-can-monitor-for-coronavirus-symptoms/. Could a Fitbit detect coronavirus? Scientists launch mobile app to track people's heart rates and activity and link them to Covid-19 cases https://www.dailymail.co.uk/news/article-8553851/Fitbit-wearers-asked-join-study-smartwatches-detect-signs-coronavirus.html.

74 Coronavirus antibody tests could do more harm than good by offering false hope, review warns https://www.telegraph.co.uk/news/2020/06/25/antibody-tests-could-do-harm-good-giving-people-false-hope-protected/.

75 “Immunity passports" in the context of COVID-19 https://www.who.int/news-room/commentaries/detail/immunity-passports-in-the-context-of-covid-19.

76 Back to life, back to normality: https://www.immunitypassport.co/home See also, The US government is in talks with AI startup Onfido to roll out immunity passports. https://www.businessinsider.com/coronavirus-onfido-immunity-passports-2020-4?international=true&r=US&IR=T. See also, Controversial ‘immunity passports’ could rely on facial recognition technology https://tech.newstatesman.com/coronavirus/controversial-immunity-passports-could-rely-on-facial-recognition-technology.

77 The UK government is in talks with facial recognition firms to develop COVID-19 immunity passports https://www.businessinsider.com/coronavirus-uk-in-talks-with-id-startups-over-immunity-passports-2020-4?international=true&r=US&IR=T.

78 COVID-19 Credentials Initiative. https://www.covidcreds.com/.

79 India's digital ID system deepens exclusion of vulnerable communities amid pandemic https://globalvoices.org/2020/06/29/marginalizedaadhaar-digital-identity-in-the-time-of-covid-19/.

80 Indonesia clamping down on fake medical certificates used to circumvent COVID-19 travel curbs https://www.channelnewsasia.com/news/asia/indonesia-covid-19-fake-medical-certificates-bali-travel-ban-12748770.

81 Facial Recognition Firms Pitch COVID-19 ‘Immunity Passports’ For America And Britain https://www.forbes.com/sites/thomasbrewster/2020/05/20/facial-recognition-firms-pitch-covid-19-immunity-passports-for-america-and-britain/#6243aa9a5914.

82 Tony Blair makes the case for a digital ID scheme (again) in a post-COVID-19 world https://diginomica.com/look-i-could-be-wrong-about-tony-blair-makes-case-digital-id-scheme-again-post-covid-19-world.

83 COVID-19 ‘Immunity Passport’ Unites 60 Firms on Self-Sovereign ID Project https://sg.finance.yahoo.com/news/covid-19-immunity-passport-unites-140503280.html.

84 Apple And Google Admit Ethereum App To Let Employees Prove They’ve Been Vaccinated. https://www.forbes.com/sites/michaeldelcastillo/2020/06/16/apple-and-google-admit-ethereum-app-to-let-employees-prove-theyve-been-vaccinated/#68a0a27d40f7.

85 Advisor resigns from ID2020 objecting to blockchain immunity passports for COVID-19 https://www.ledgerinsights.com/id2020-resignation-blockchain-covid-19-immunity-passports/.

86 De wereld heeft een nieuw besturingssysteem nodig. https://www.nrc.nl/nieuws/2020/06/19/de-wereld-heeft-een-nieuw-besturingssysteem-nodig-a4003444.

87 'The surge of sensationalist COVID-19 AI research’ https://venturebeat.com/2020/04/24/the-surge-of-sensationalist-covid-19-ai-research/.

88 ‘UK government using confidential patient data in coronavirus response’ https://www.theguardian.com/world/2020/apr/12/uk-government-using-confidential-patient-data-in-coronavirus-response.

89 ‘South Korea to adopt wristbands for quarantine violators’ https://asia.nikkei.com/Spotlight/Coronavirus/South-Korea-to-adopt-wristbands-for-quarantine-violators2.

90 ‘COVID-19 ‘Immunity certificates’: practical and ethical conundrums’ https://www.statnews.com/2020/04/10/immunity-certificates-covid-19-practical-ethical-conundrums/.

91 Securitytest potentiële Corona-apps https://www.rijksoverheid.nl/binaries/rijksoverheid/documenten/publicaties/2020/04/19/rapportage-veiligheidstest-potentiele-corona-apps/Finale+rapportage+Ministerie+van+VWS+Corona+Apps+19042020+definitief.pdf.

92 'Your Boss May Soon Track You At Work For Coronavirus Safety’ https://www.npr.org/2020/05/08/852896051/your-boss-may-soon-track-you-at-work-for-coronavirus-safety/.

93 ‘Screen New Deal' https://theintercept.com/2020/05/08/andrew-cuomo-eric-schmidt-coronavirus-tech-shock-doctrine/.

94 ‘Vestager: It’s not a choice between fighting the virus and protecting privacy’. https://www.euractiv.com/section/digital/news/vestager-its-not-a-choice-between-fighting-the-virus-and-protecting-privacy/.

95 'More scary than coronavirus': South Korea's health alerts expose private lives. https://www.theguardian.com/world/2020/mar/06/more-scary-than-coronavirus-south-koreas-health-alerts-expose-private-lives.

96 'Show evidence that apps for COVID-19 contact-tracing are secure and effective’ https://www.nature.com/articles/d41586-020-01264-1.

97 'Do we need more coronavirologists in the pandemic debate?' https://www.timeshighereducation.com/news/do-we-need-more-coronavirologists-pandemic-debate.

98 TPM: Comprehensive engineering https://www.tudelft.nl/tbm/onderzoek/tpm-comprehensive-engineering/.

99 Wie die Pandemie die EU-Digitalpolitik entzaubert https://www.republik.ch/2020/08/04/wie-die-pandemie-die-eu-digitalpolitik-entzaubert.

100 “Australia's top coronavirus adviser at the World Health Organisation says she won't use it due to lingering privacy concerns.”. https://www.smh.com.au/politics/federal/half-of-us-say-we-support-the-COVIDsafe-app-but-only-16-per-cent-have-downloaded-it-20200501-p54p53.html.
